# A Guided Wave Sensor Based on the Inverse Magnetostrictive Effect for Distinguishing Symmetric from Asymmetric Features in Pipes

**DOI:** 10.3390/s150305151

**Published:** 2015-03-02

**Authors:** Jiang Xu, Xinjun Wu, Dongying Kong, Pengfei Sun

**Affiliations:** School of Mechanical Science and Engineering, Huazhong University of Science and Technology, Wuhan 430074, China; E-Mails: xinjunwu@mail.hust.edu.cn (X.W.); kongdongying@163.com (D.K.); hustpfs@mail.hust.edu.cn (P.S.)

**Keywords:** guided wave sensor, inverse magnetostrictive effect, flexural-mode wave, asymmetric feature, pipe

## Abstract

The magnetostrictive guided wave sensor with a single induced winding cannot distinguish axially symmetric from non-axially symmetric features in a pipe, because it is impossible for the sensor to detect the non-axially symmetric mode waves. When we study the effect of the change of the magnetic field in the air zone for receiving the longitudinal guided wave mode, we find that the change of the magnetic flux in the air zone is almost equivalent to the change of the flux in the pipe wall, but in opposite directions. Based on this phenomenon, we present a sensor that can detect the flexural-mode waves in pipes based on the inverse magnetostrictive effect. The sensor is composed of several coils that are arranged evenly on the outside of pipes. The coils induce a change in magnetic flux in the air to detect the flexural-mode waves. The waves can be determined by adding a phase delay to the induced signals. The symmetric and asymmetric features of a pipe can be distinguished using the sensor. A prototype sensor that can detect F(1,3) and F(2,3) mode waves is presented. The function of the sensor is verified by experiments.

## 1. Introduction

The guided wave technique has become successful in nondestructive testing (NDT) over the last decade because guided elastic waves can propagate over a long distance and may be excited and received using transducers positioned at a given location [1,2,3,4,5]. Guided waves can propagate along cylinders over a long distance without critical energy density loss due to the closure of the walls in circumference, which is a very important aspect of the NDT method for pipelines [6,7,8]. The axially symmetric L(0,2) and T(0,1) modes are the most widely-used modes for pipelines. The L(0,2) mode is practically non-dispersive over typical frequency ranges, and particle motion is roughly uniform throughout the pipe wall. The T(0,1) mode is non-dispersive across the entire range of frequencies used, and its characteristics are not affected by the presence of liquid in a pipe, which is favorable for inspecting pipelines in service.

In applications, echoes occur not only at corrosion defects, but at other features, as well, such as welds, flanges and square ends. The reflection signal from flanges and square ends is generally bigger than the defect signal. All of the energy in the signal is reflected from flanges and square ends. We can identify flanges and square ends from the amplitude of the signal. However, the reflection from a butt weld is small, because the weld cap and weld bead present only a small change to the geometry. This phenomenon introduces the possibility of a weld being incorrectly identified as a defect. In practical testing, the pipeline maintainer sometimes provides the structural drawings of the pipeline and sometimes does not. The reasons are that their original design drawing is missing and the pipeline is modified without providing the structural drawings, which lead to detecting the pipeline without structural drawings. The axially symmetric mode is reflected when the axially symmetric mode is incident on an axially symmetric feature. In addition, non-axially symmetric waves will be generated when a feature is non-axially symmetric. It is difficult to identify these features and defects from axially symmetric reflections. The mode conversion induced by non-axially symmetric features can be measured to overcome this problem [9,10,11,12,13,14,15]. If the L(0,2) mode is incident, the main conversion modes are the F(1,3) and F(2,3) modes. If the T(0,1) mode is incident, the mode conversion is primarily to the F(1,2) mode. The extent to which a mode is converted to a reflection can be applied to estimate the symmetry of different features.

Many methods have been employed for generating and detecting guided waves in pipes. Piezoelectric technology and magnetostrictive technology are the most widely used [1,3]. Piezoelectric sensors are constructed using many piezoelectric elements, e.g., 16, 32 and 64 elements. The structure of the sensors allows for axially symmetric modes and non-axially symmetric modes to be easily detected by adding a phase delay to the signals of the piezoelectric elements [14]. To generate pure symmetric modes, the performance of transducers must be consistent. Magnetostrictive transducers are simpler and less expensive than piezoelectric transducers for generating and detecting the non-axially symmetric guided wave mode in pipes. It is impossible to obtain non-axially symmetric modes because magnetostrictive sensors do not have circumferential resolution [13]. Therefore, magnetostrictive sensors cannot distinguish axially symmetric from non-axially symmetric features in pipes. However, the symmetry of features is a very important parameter in classifying the severity of defects. Recently, Vinogradov *et al.* reported the segmented MsT (magnetostrictive transducer), which was based on several AC windings wrapped through the strip [16,17,18,19,20,21]. Each winding induced a change in magnetic flux in a segment of the strip to detect the guided waves. This sensor could obtain the flexural mode waves based on the phase delay method [14]. Since the pipe is an integral whole in the circumference direction, the magnetic field in the pipe wall could not be divided as several segments.

When we recently studied the effect of the change in the magnetic field in the air zone for receiving guided waves based on the inverse magnetostrictive effect, we observed that the change in the magnetic flux in the air zone was nearly equivalent to the change in the flux in the pipe wall, but along the opposite direction [22]. It is simple to divide the magnetic field in the air zone along the circumferential direction. Based on this phenomenon, a guided wave sensor based on the inverse magnetostrictive effect for distinguishing symmetric from asymmetric features in pipes was developed. The sensor features several coils that are arranged evenly on the outside of pipes. The coils induce a change in the magnetic flux in the air zone to detect the guided waves in pipes. Non-axially symmetric mode waves can be obtained by adding a phase delay to the induced signals. The axially symmetric and non-axially symmetric features of a pipe can thus be distinguished using the sensor, as verified by experiments.

## 2. Operating Principle of the Sensor

When a ferromagnetic material is subjected to a change in stress during magnetization, a change in the magnetization of the material is observed. This phenomenon is defined as the inverse magnetostrictive effect. In addition, the longitudinal inverse magnetostrictive effect is called the Villari effect, and the torsional inverse magnetostrictive effect is called the Matteucci effect. Based on the principle of energy conservation, the relation between the mechanical and magnetic properties of a material can be expressed as follows:

(1) $$B=dT+\mu^{T}H$$

where *B* is the flux density, *d* is the magnetostrictive cross-coupling coefficient, *T* is the stress, *μ^T^* is the permeability at constant *T* and *H* is the magnetic field strength. The piezomagnetic equation is normalized in IEEE Standard 319–1990 [23]. The equation gives the relation between the stress and the magnetic flux density as the inverse magnetostrictive effect. When guided waves propagate along pipes, the stress in the corresponding position varies. A change in the magnetic flux density is exhibited in the pipe wall. The receiving coil encircling the pipe induces a change in magnetic flux to receive guided waves. The waves of non-axially symmetric modes cannot be obtained, because the coil induces a change in the overall magnetic flux.

**Figure 1:**
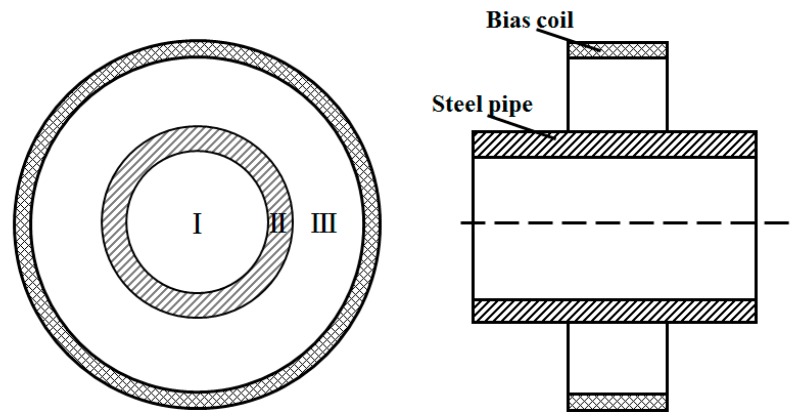
The layout of the receiving position. Zone I: the air zone inside the pipe; Zone II: the wall of the pipe; Zone III: the air zone from the outside surface of the pipe to the bias coil.

In the radial direction, the pipe is divided into three regions, as shown in Figure 1. When guided waves propagate along the pipe, the stress in the corresponding position varies in Region II. A change in the magnetic flux density occurs based on the inverse magnetostrictive effect. Therefore, the variation of the magnetic field can be measured to detect guided waves. An ordinary magnetostrictive sensor is a solenoid coil or a ribbon-encircled coil. When waves propagating through pipes are detected from the outside, the output signal can be expressed as follows:

(2) $$V_{III}\left(r_{2},t\right)=\frac{nd(\varphi_{II}(t)+\varphi_{I}(t)+\varphi_{III}(r_{2},t))}{dt}$$

where *r*_2_ is the radius of the coil and *r*_2_ is larger than the outer radius of the pipe. As previously indicated, waves of non-axially symmetric modes cannot be detected, because the coil induces a change in the overall magnetic flux. Therefore, the sensor cannot use the mode conversion method to distinguish symmetric from asymmetric features in a pipe.

In our recent study, we reported that the change in the flux in air is opposite of the change in the flux in the pipe wall [22]. Furthermore, the waveform of the induced signal in the air zone is nearly the same as that in the pipe wall. The sensor can induce a change in the flux in the air zone to receive guided waves based on the inverse magnetostrictive effect. It is well known that the magnetic field in air Zone III can be divided easily along the circumferential direction. If the sensor had *N* coils to induce a change in the flux in air Zone III, non-axially symmetric mode waves could be detected as piezoelectric sensors. The induced signal of each coil would be:

(3) VN(t)=ndφNIII(r2',t)dt

Based on the method described in Lowe’s paper [14], axially and non-axially symmetric modes can be detected using the induced signals.

## 3. Prototype Sensor

**Figure 2:**
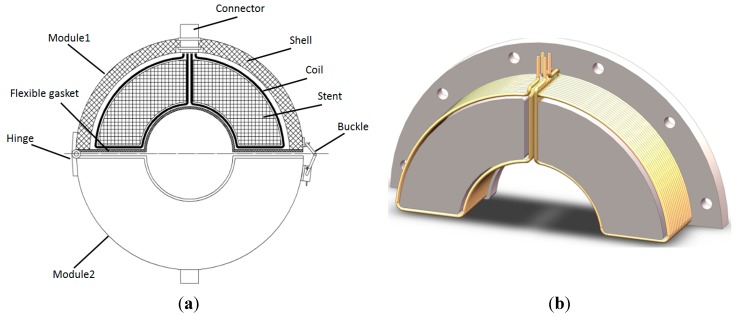
Schematic diagram of the prototype sensor used to obtain the F(1,3) and F(2,3) modes. (**a**) The sensor structure; (**b**) the coil structure in each module.

Based on the above-described analysis and considering the ease of use, a sensor with a detachable structure was designed. The sensor consists of two of the same modules that are connected by a hinge and buckle, as shown in Figure 2a. The module features several coils, a stent, a shell, a connector and a flexible gasket. The number of coils is determined by the circumferential order of the flexural modes. If we want to obtain the *n*-th circumferential order of flexural modes, each module should feature *n* coils based on the structure of the flexural mode waves. Each coil covers a sector of *π/n* degrees in Zone III. To obtain F(1,3) and F(2,3) modes, each module should feature at least two coils. Therefore, the prototype sensor consists of two modules. Each module contains two coils, and each coil covers a sector of π/2 degrees to induce a change in magnetic flux in Zone III. The coils encircle the convex platform of the stent, as shown in Figure 2b. To prevent the sensor from affecting the received signal, the stent and the outer shell are made of Teflon or nylon.

In designing the sensor, the parameters of the coil must be considered, such as the number of turns, width, inner radius and outer radius. Moreover, the size of the stent and the outer shell should be modified according to these parameters. As in the case of the ordinary magnetostrictive guided wave sensor, a large number of turns will help improve the amplitude of the induced signal, and the width of the coil should be less than half of the detected wavelength. The inner radius of the coil should be made similar to that of the pipe wall, because the contribution of the change in the magnetic flux in Zone III is greater close to the pipe wall. The outer radius, on the other hand, should ideally be infinite, which is, however, impossible. In addition, the space between the coils and the bias magnetizer must be suitable for receiving guided waves.

## 4. Experimental Verification

### 4.1. Experimental Setup

**Figure 3:**
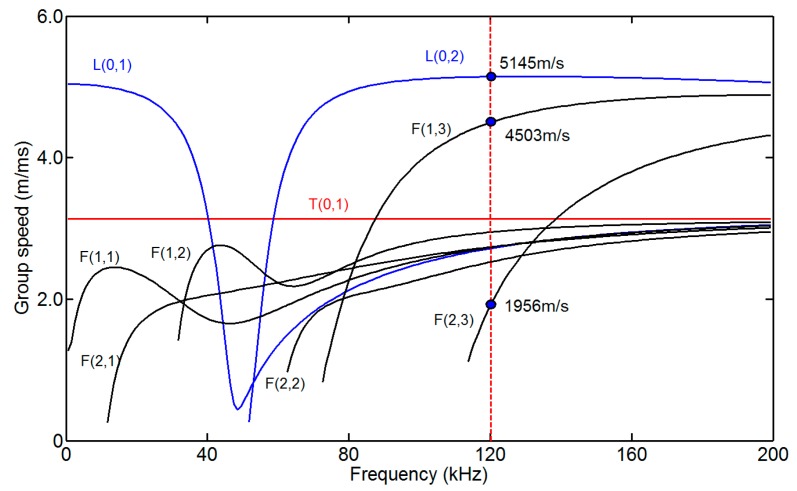
Group speed dispersion curves of the pipe with a 38-mm outside diameter and 5-mm wall thickness.

**Figure 4:**
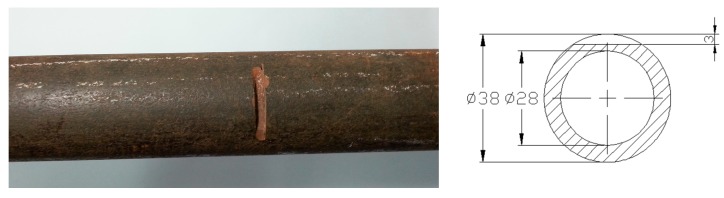
The photo and the schematic diagram of the notch.

**Figure 5:**
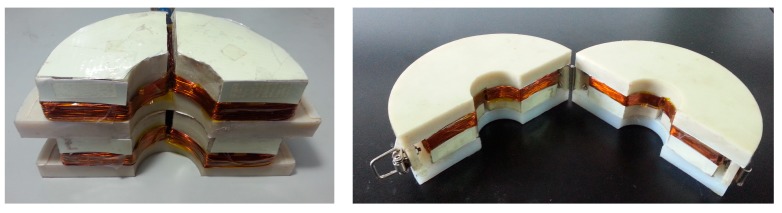
The photos of the prototype sensor for the pipe with a 38-mm outside diameter.

**Figure 6:**
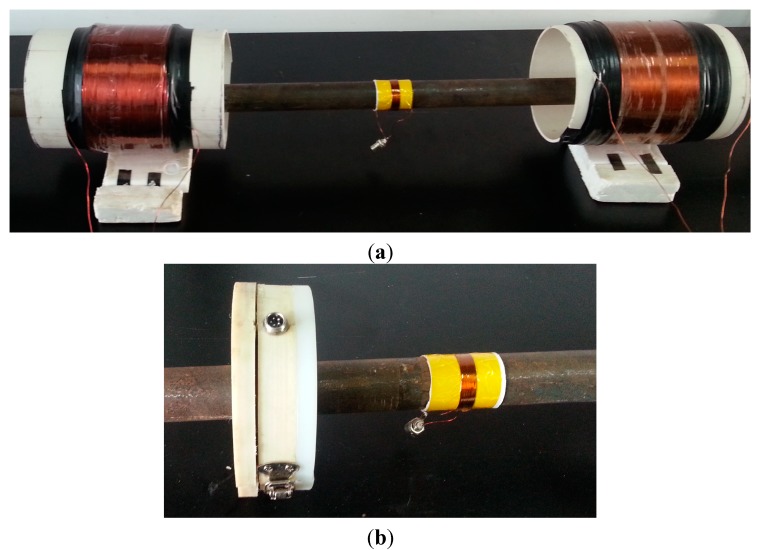
The photos of the sensors (**a**), the excitation coil and two bias coils (**b**), as well as the conventional receiving sensor and the prototype receiving sensor.

**Figure 7:**
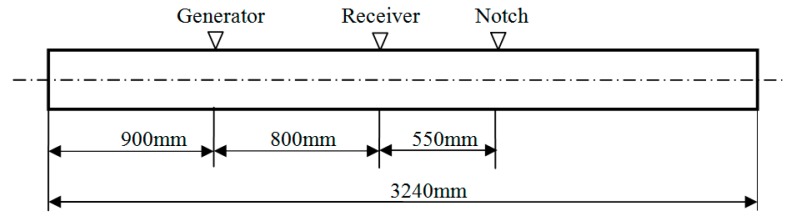
Schematic diagram of the experimental setup.

A low-carbon steel pipe with an outer diameter of 38 mm, a wall thickness of 5 mm and length of 3.24 m was employed as the specimen. The dispersion curves of the pipe are shown in Figure 3, which were calculated using the DISPERSE software program [24]. To reduce the effect of dispersion, the excitation frequency of L(0,2) was set to 120 kHz. A notch 3 mm deep and 3 mm wide was machined 990 mm from the near end of the pipe. The photo and the schematic diagram of the notch are shown in Figure 4. The stent and the outer shell were made of nylon. A sensor composed of four coils (Coil 1, Coil 2, Coil 3 and Coil 4) was fabricated for the pipe. The photos of the sensor are shown in Figure 5. The four coils were made of No. 25 gauge wire with a single layer, 20 turns, a width of 10 mm, an inner radius of 20 mm in a fan shape and an outer radius of 74 mm in a fan shape. The basis for selecting the parameters of the coils is as follows. When the width of the induced coil is equal to half the wavelength of the wave, the amplitude of the induced signal is the maximum value generally. Here, the wavelengths of L(0,2), F(1,3) and F(2,3) at 120k Hz are 42.875 mm, 37.5 mm and 16.3 mm, respectively. Based on the wavelengths of three modes, the width of the receiving coil was selected as 10 mm. The inner diameter of a fan shape was selected based on the outer diameter of the pipe. The outer diameter of a fan shape was selected based on the inner diameter of the bias coil. The excitation coil was made of No. 25 gauge wire with a single layer, 20 turns, a width of 10 mm width and a diameter of 38.5 mm. The excitation coil was encircled outside the pipe. To compare the signals received by the aforementioned sensor and the ordinary sensor, an ordinary sensor (Coil A) identical to the excitation coil was used. The bias coils on the excitation side and on the receiving side, which were made of No. 16 gauge wire with 5 layers, 500 turns, a length of 177 mm and a diameter of 200 mm, were the same. The photo of the excitation coil and two bias coils is shown in Figure 6a, and the photo of the ordinary receiving coil and the prototype sensor on the pipe is shown in Figure 6b. The direct current of the bias coils was 3.3 A, which provided a sufficiently strong axial bias field to make the magnetostriction effect linear. A schematic diagram of the experimental setup is shown in Figure 7. The center of Coil 1 coincided with the center of the notch in the circumferential direction of the pipe. The excitation signal was a 4-cycle tone burst at 120-kHz center frequency modified by a Hanning window with a peak to peak voltage of 200V. The signals induced by the receiving coils were amplified (by approximately 80 dB). The signals were subsequently digitized using a multi-channel A/D card, which operates at a sample speed of 2 MS/s (mega-samples per second) per channel. Digitization was repeated 300 times to reduce white noise. 

### 4.2. Experimental Results

**Figure 8:**
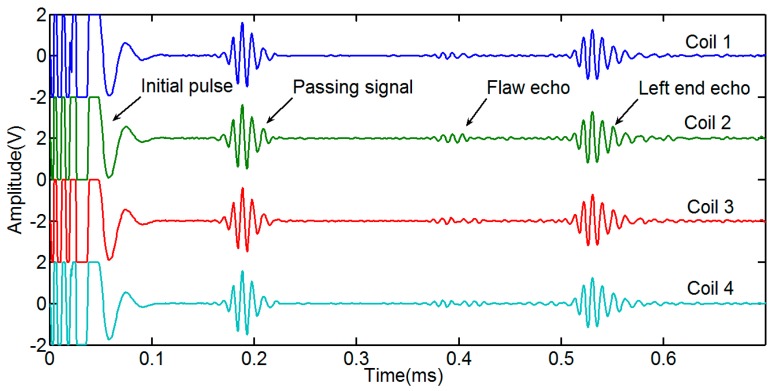
Receiving signals induced by the four coils of the prototype sensor.

**Figure 9:**
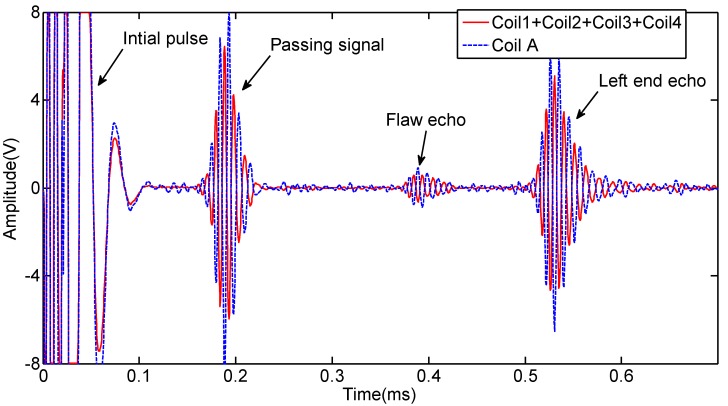
Added signal induced by the four coils (induced Zone III) and the signal induced by Coil A (induced Zone II + Zone I).

The signals received by the four coils are shown in Figure 8. The waveforms of the four passing signals are similar. The waveforms of four flaw echoes are different in the time of duration and the amplitude. The reason is that the flaw echoes include not only the L(0,2) mode, but also the flexural mode. We added the four signals directly to obtain the L(0,2) mode based on Lowe’s method [14], which is shown in Figure 9. The signal induced by an ordinary sensor (Coil A) is also shown in Figure 9. The signal induced by Coil A with the added signal induced by the four coils is nearly out of phase. The amplitude of the Coil A signal is greater than that of the added signal.

**Figure 10:**
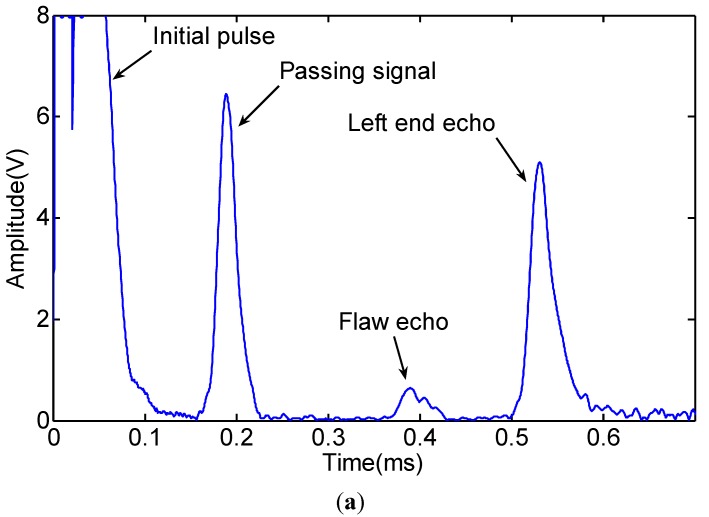

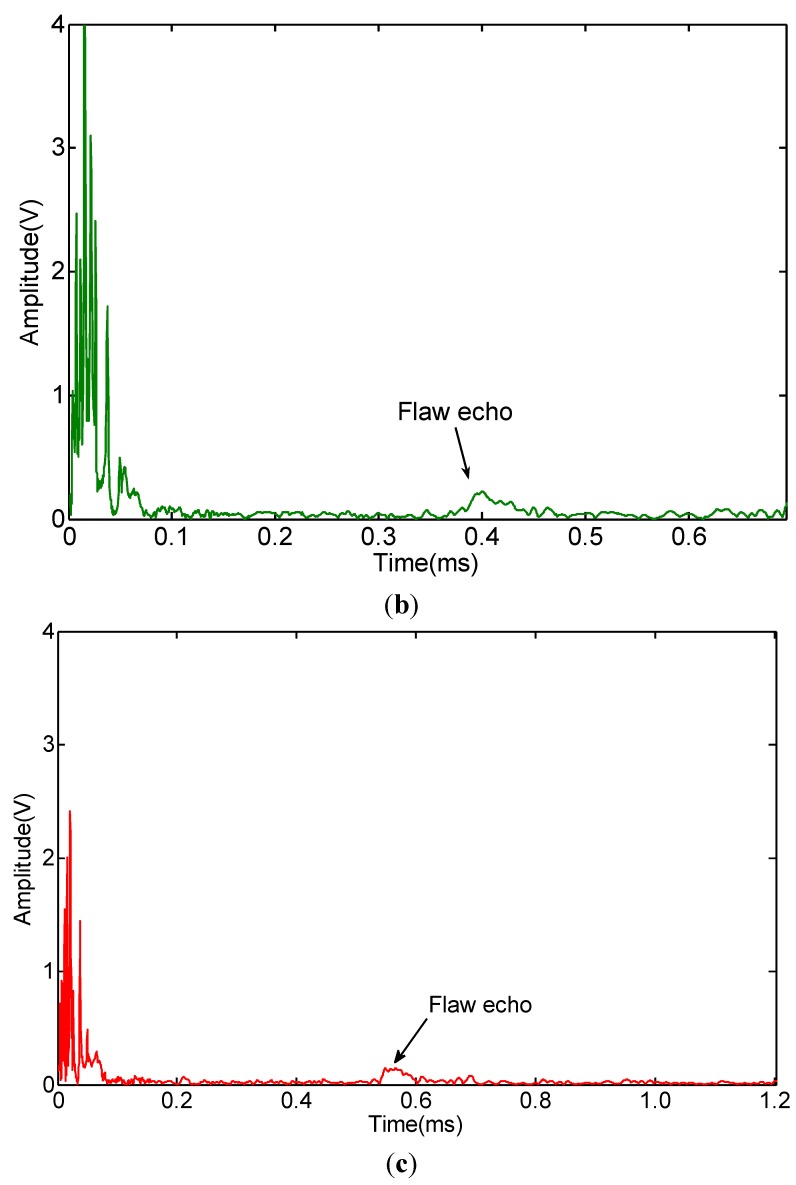
Waveform of the axially and non-axially symmetric modes obtained by processing the data shown in Figure 8: (**a**) L(0,2) mode; (**b**) F(1,3) mode; (**c**) F(2,3) mode.

To obtain non-axially symmetric modes, we added a phase delay to the four signals [14]. The signal process is as follows. First, we process the four signals using Hilbert transform. Second, the transformed signals multiply the phase delay. To obtain the F(1,3), the real part of the transformed signals multiplies cos(0), cos(π/2), cos(π), cos(3π/2), and the imaginary part of the transformed signals multiplies sin(0), sin(π/2), sin(π), sin(3π/2). To obtain the F(2,3), the real part of the transformed signals multiplies cos(0), cos(2 × π/2), cos(2 × π), cos(2 × 3π/2), and the imaginary part of the transformed signals multiplies sin(0), sin(2 × π/2), sin(2 × π), sin(2 × 3π/2). Last, the Hilbert transformed signals of F(1,3) and F(2,3) are obtained by taking the absolute value of the processed signals. The signals of the L(0,2) F(1,3) and F(2,3) mode are shown in Figure 10. We take the notch as the non-axially symmetric feature and the left end as the axially symmetric feature. To study whether the non-axially symmetric mode echo from the left end, the time axial of the F(2,3) mode is extended to 1.2 ms based on the wave traveling time. The initial pulse signals of F(1,3) and F(2,3) are disordered and useless for being tested, which will be ignored in the following analysis. As shown in Figure 10a, the passing signal only includes the L(0,2) mode, and the arrival time is about 0.156 ms, which is consistent with the theoretical calculation result. Based on the dispersion curves of the pipe and the experimental setup, we can infer that the arrival time of the L(0,2) mode flaw echo is approximately 0.369 ms (travel distance 1.9 m at 5145 m/s); that of the F(1,3) mode flaw echo is approximately 0.385 ms (travel distance 1.35 m at 5145 m/s and travel distance 0.55 m at 4503 m/s); and that of the F(2,3) mode flaw echo is approximately 0.544 ms (travel distance 1.35 m at 5145 m/s and travel distance 0.55 m at 1956 m/s). The flaw echo arrival times of the L(0,2) F(1,3) and F(2,3) modes are 0.371 ms, 0.386 ms and 0.541 ms from Figure 10, respectively. If there are F(1,3) and F(2,3) modes reflected from the left end, the echo arrival time of the F(1,3) and F(2,3) modes are 0.552 ms (travel distance 0.9 m at 5145 m/s and travel distance 1.7 m at 4503 m/s) and 1.044 ms (travel distance 0.9 m at 5145 m/s and travel distance 1.7 m at 1956 m/s), respectively. There are no non-axially symmetric mode echoes from the left end, as shown in Figure 10b,c. Therefore, the given sensor could receive non-axially symmetric modes to distinguish symmetric from asymmetric features in the pipe.

## 5. Conclusions and Future Work

A guided wave sensor based on the inverse magnetostrictive effect is developed to distinguish symmetric from asymmetric features in pipes. The sensor features several coils that are arranged evenly on the outside of pipes. The coils induce a change in the magnetic flux in the air zone out of pipes to receive guided waves. Non-axially symmetric mode waves can be obtained by adding a phase delay to the induced signals. The axially symmetric and non-axially symmetric features of pipes can be distinguished using the non-axially symmetric guided wave mode. In a future study, we will use the sensor developed herein to carry out the defect circumferential quantitative method in pipes and detect defects at the same location as welds or flanges.
